# A System-Level Methodology for the Design of Reliable Low-Power Wireless Sensor Networks

**DOI:** 10.3390/s19081800

**Published:** 2019-04-15

**Authors:** Oussama Brini, Dominic Deslandes, Frederic Nabki

**Affiliations:** Department of Electrical Engineering, École de Technologie Supérieure, 1100 rue Notre-Dame Ouest, Montréal, QC H3C 1K3, Canada; dominic.deslandes@etsmtl.ca (D.D.); frederic.nabki@etsmtl.ca (F.N.)

**Keywords:** wireless sensor network (WSN), design methodology, energy model, path-loss, low-power, system-level design, link layer, automatic repeat request (ARQ), forward error correction (FEC)

## Abstract

Innovative Internet of Things (IoT) applications with strict performance and energy consumption requirements and where the agile collection of data is paramount are arising. Wireless sensor networks (WSNs) represent a promising solution as they can be easily deployed to sense, process, and forward data. The large number of Sensor Nodes (SNs) composing a WSN are expected to be autonomous, with a node’s lifetime dictated by the battery’s size. As the form factor of the SN is critical in various use cases, minimizing energy consumption while ensuring availability becomes a priority. Moreover, energy harvesting techniques are increasingly considered as a viable solution for building an entirely green SN and prolonging its lifetime. In the process of building a SN and in the absence of a clear and well-rounded methodology, the designer can easily make unfounded and suboptimal decisions about the right hardware components, their configuration, and reliable data communication techniques, such as automatic repeat request (ARQ) and forward error correction (FEC). In this paper, a methodology to design, configure, and deploy a reliable ultra-low power WSNs is proposed. A comprehensive energy model and a realistic path-loss (PL) model of the sensor node are also established. Through estimations and field measurements it is proven that, following the proposed methodology, the designer can thoroughly explore the design space and the make most favorable decisions when choosing commercial off-the-shelf (COTS) components, configuring the node, and deploying a reliable and energy-efficient WSN.

## 1. Introduction

Wireless sensor networks (WSNs) are increasingly being deployed in a broad range of applications, such as home automation [1,2], smart cities [3,4], industrial automation [5,6,7,8,9,10], and precision agriculture [11,12,13]. This is because WSNs are low cost and composed of easy to deploy battery-operated devices. However, as a small form factor is also an important requirement, the use of small batteries hinders the operation of WSNs for several years without replacing or recharging them. Accordingly, various energy harvesting (EH) techniques are considered a viable green solution for powering sensor nodes (SNs) [14,15,16,17,18]. In this case, rechargeable energy buffers (e.g., supercapacitors) are used for energy storage [19,20]. Renewable energy sources, such as vibration, light, or heat can be considered for powering a SN when several harvesters are used and a proper dimensioning of the energy buffer is carried-out. Consequently, the research community’s first goal has always been to find both hardware [21,22,23] and software [24,25,26] solutions to decrease the depletion rate of the aforementioned limited energy sources.

Therefore, a significant amount of research work has been undertaken in order to estimate the power consumption and the energy consumption of WSNs at an early stage of the design process [27,28]. For instance, a power/energy estimator is presented in [29], allowing for the prediction of a WSN’s autonomy in order to evaluate the economic benefits of replacing an existing wired network with a wireless one. The hardware power consumption models are determined using the functional level power analysis (FLPA) methodology [30]. In addition, multiple energy harvesting systems are considered (i.e., solar, wind, and thermal). Dynamic Power Management (DPM) is performed according to a finite state machine (FSM), where the transitions are dictated by the energy saving levels and weather forecasts. Additionally, the authors in [31] present an abstract modelling framework for both sensor-network-level and sensor-node-level modelling and apply a hardware/software co-design approach. The framework is based on SystemC and can be used to model almost all of the aspects from sensors’ modes of operation to radio signal propagation. Moreover, at the sensor-node-level, the model is split into two different but tightly dependent and related sections (i.e., software and hardware section). The hardware section helps the estimation of the sensor node’s overall power consumption by monitoring the significant parameters of the model, while the software section, on the other hand, comprises tasks models such as processing, I/O tasks, services, and scheduling of a real time operating system (RTOS) model. It helps simulating the functional side, such as the behavior of contention-based medium access control (MAC) protocols [32]. At the sensor-network-level, that work models the physical phenomenon of the environment where the sensor node’s hardware model will be integrated. 

Another interesting contribution is the Powersim C++ class library presented in [33]. It monitors the C++ operators during the simulation of a high-level of abstraction model developed using SystemC in order to estimate a given hardware’s power consumption when provided with an energy model. An energy model represents a set of simulated or hardware power consumption measurements of different operators and it is possible for the designer to choose the modules and operators to be monitored by adding a configuration file. The energy model contains a list of energy granularities of each arithmetic and logic operation supported by a given microcontroller unit (MCU). This way, Powersim can calculate the overall energy consumption of the algorithm. The same code was then ported to an MCU and measurements showed that the simulation results with Powersim present an error of 15.8%.

In addition, work presented in [34] uses the Stateflow graphical modeling environment [35], which is a component of Simulink, to develop a model-based design framework of an energy-optimized protocol stack for WSNs. It allows the simulation and code generation of WSN applications intended for a variety of implementation platforms. While omitting the code generation capability of the tool, a model-based approach for the design of ultra-low power wireless sensor nodes, along with a high-level of abstraction modeling framework based on Stateflow, is also introduced in [36]. This work is a continuation of the latter. Presently, the comprehensive energy and power model can lead to a fast and effective method of designing low-power wireless sensing systems by serving as a guideline for choosing the right COTS components and node configuration.

Moreover, given that a SN is intended to operate within a large group of other SNs, having a realistic model for path loss (PL) in order to estimate operating range is of interest. The authors in [11] model the signal PL between two endpoints acting in an open space such as a rural field by taking into account the line-of-sight (LOS) component and the reflected signal due to the ground. In [37], a three-slope log-normal path loss model was proposed in order to model a narrowband radio channel in rural scenarios where the radios operate under near-ground conditions, such as is the case for smart agriculture applications. Moreover, a survey of LOS and non-line-of-sight (NLOS) wireless PL models is presented in [38]. For LOS links, a fitting factor is added to the free-space PL in an attempt to avoid underestimations. 

In this work, we proposed a PL model that more accurately estimates signal attenuation in the sub-1 GHz ISM band. Based on previous works in the literature [11,38] and field measurements, it takes into account the free-space PL, ground reflection, and a fitting factor with a variable coefficient. In addition to accurate energy and range estimations, this work also focuses on finding the energy-latency-reliability trade-off, which is very important in WSN applications since it captures the interdependence of key parameters from a quality of service (QoS) point of view. 

To ensure reliable data communication, automatic repeat request (ARQ) protocols [39] and forward error correction (FEC) techniques [40,41], or a combination of both [42,43,44], are mostly used. In this analysis, a simple point-to-point communication link is considered. Readers can consult [8,42] in order to further understand the effects of the used medium access protocol, multi-hop routing, and the broadcast nature of the WSN, which are beyond the scope of this work.

Moreover, recently, research on limiting energy consumption while meeting stringent QoS requirements has taken a considerable leap, especially in industrial automation applications [5,8,45].

Work in [46] investigates the adaptive data rate algorithm implemented in long range wide area network (LoRaWAN) and its theoretical bounds of link and network capacity. The work explains how the data rate is dynamically adjusted such that a node close to the gateway would use a small spreading factor in order to increase the raw data rate and be able to decrease latency and radio output power. Therefore, the closest node to the gateway transmits with the maximum data date and lowest output power. In LoRaWAN compliant devices, the adaptive rate-power allocation is based on received signal strength indicator (RSSI) and signal-to-noise readings of the last received packets of static devices. As it will be demonstrated in this work, the studied power/data rate allocation technique used in LoRaWAN can drastically enhance the link performance and energy efficiency.

While considering that, in harsh industrial application scenarios, factors such as transmission power level, communication range, and random ambient noise affect radio link quality, a network-level reliability model for estimating and increasing the reliability performance and deployment parameters of industrial WSN is presented in [5]. The work suggests a new approach where nodes measure and estimate link parameters, such as the packet reception ratio (PRR) and received signal strength (RSS), and then optimize the lower-bound reliability value. To this end, a background noise model and a modified log-normal path loss model to estimate the RSS are introduced. A mapping function between the packets-received-ratio (PRR), background noise, and RSS is then proposed. Through a case study, the work demonstrates the feasibility of the solution and increases the reliability by computing the maximum deployment distance between sensor nodes. Yet, the energy consumption cost is not quantitatively evaluated and optimized as the work mentions that the nodes are energy-limited devices. Moreover, the latency is not clearly addressed.

After adjusting the wireless link parameters, bit and packet errors can still occur. Therefore, as previously mentioned, FEC, ARQ schemes, or both are used. As FEC has a limited ability to correct errors, the authors in [47] propose a dynamic error control scheme based on link parameters such as BER and ambient noise in WSN. Through simulations, it is reported that throughput and retransmission probability are improved. An energy model that showcases the efficiency of the proposed technique is not reported in that work.

As ARQ-based protocols suffer from feedback error, work in [39] studies different approaches allowing to increase feedback channel time diversity and attain different reliability regions with respect to feedback channel error rate such as the *L*-REP-ACK scheme. The latter is a modified version of the stop-and-wait (SAW) ARQ scheme. Then, the work proposes a new method of acknowledging packet delivery for retransmission protocols, which is based on backwards composite acknowledgment from multiple packets while relying on collaboration between transmitter (TX) and receiver (RX) nodes. Therefore, depending on channel quality, the scheduler of the wireless channel would be able to configure ultra-reliable communication when needed. The proposed solution does not require increasing the time diversity order of the feedback channel and, thus, does not incur energy consumption and latency overheads. Moreover, the work investigates the advantages and disadvantages of blind retransmissions and shows that, in extremely unreliable feedback channel conditions, an open-loop solution is viable, in terms of reliability, while noting the energy consumption downside. However, in none of the studied solutions does the work quantitatively evaluate the energy consumption nor the latency overheads. As in most cases, a good compromise between reliability, latency, and energy consumption needs to be found and the evaluation of these three performance metrics needs to be carried out simultaneously.

In [48], the work studies the possibility of achieving a 0.99999 packet success probability within a 1 ms latency, while bearing in mind the capacity of the network. To this end, the work avoids the reliance on imperfect and error-prone feedback channels and proposes a novel scheme based on blind retransmissions coupled with successive interference cancellation to receive the remaining non-decoded data with a low latency penalty, when compared with the feedback-based retransmission schemes. Finally, it is reported that depending on the number of users sharing the resources, the novel scheme can be more resource efficient than a conservative single shot transmission. However, the work assumes fast processing and transmitting/receiving times. This assumption can also lead to a significant communication range reduction when operating at high data rates.

The remainder of this paper is structured as follows: Section 2 presents the sensor node’s energy model and modeling framework. Section 3 explains the proposed wireless link characterization approach and presents the proposed path-loss model. Section 4 describes how the achievable data rate can be estimated. Section 5 presents the theory behind the reliability performance of different data transfer techniques. Section 6 summarizes the methodology and includes two case studies where the reliability-latency-energy trade-off is highlighted. Finally, Section 7 concludes the paper.

## 2. Sensor Node Energy Model

In this section, the analytical energy estimation models and the modeling framework of a functional sensor node are introduced. The considered sensor measures the temperature, pressure, and humidity. At the end of the section, a comparison between the estimated and the measured energy per measurement is given.

### 2.1. Modeling Framework

**Figure 1:**
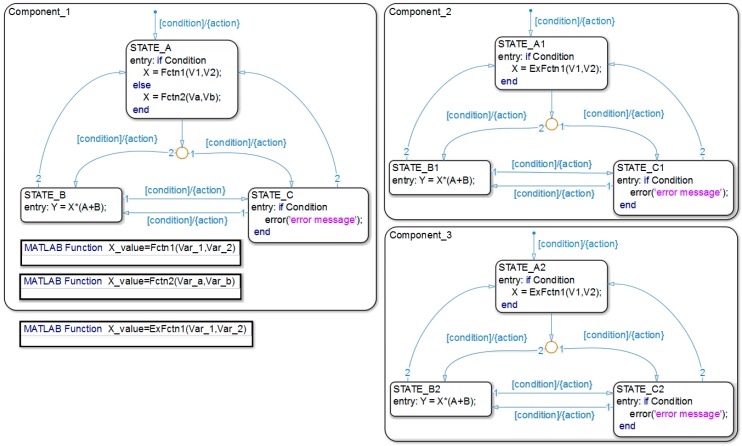
A general sensor node energy model using Stateflow charts.

The presented modeling framework [36] is based on Simulink/Stateflow and allows the creation of energy consumption models of configurable COTS components, based on FSMs. The main components of a SN taken into consideration are the microcontroller unit (MCU), the transceiver, and the sensor. A Stateflow chart functions as an FSM within the Simulink model. In addition, it is possible to integrate MATLAB functions that can reside anywhere in a Stateflow chart, state, or sub-chart. Therefore, the framework ensures a high degree of modeling flexibility, as shown in Figure 1. It also shows that it represents multiple levels of subcomponents in a system, making multilevel-state complexity of a SN more manageable.

### 2.2. Energy Model Parameters

In Table 1, all the parameters that have an impact on the overall energy consumption of the studied SN, based on the CC1310 system-on-chip (SoC) [49], are listed. It should be noted that only the parameters linked to the sensor considered (i.e., Bosch Sensortec BME280 [50]) are specific to this SN. Otherwise, the list of parameters can be used and adjusted to estimate the energy consumption of approximately any wireless sensing system.

### 2.3. Analytical Energy Model

#### 2.3.1. Micro-Controller Unit (MCU)

An MCU’s central processing unit (CPU) core speed and current consumption can be assessed by running a benchmark algorithm. Several benchmarking algorithms have emerged, such as Fibonacci, Dhrystone, and CoreMark (CM) [51]. The latter was developed by the Embedded Microprocessor Benchmark Consortium (EEMBC) in 2009 and then quickly became the de facto standard for CPU core performance ratings. Most MCU manufacturers specify the current consumption of their products when running one or more benchmark algorithms, notably the industry standard CM, which is the benchmark considered in this work. This is important as the designer needs a guideline for estimating a specific application’s power consumption, which can vary considerably from one benchmark algorithm to another [36]. Moreover, CM is an open-source portable program allowing designers to extract the current consumption of any MCU on the market when it is not provided by the manufacturer. Its source code is written in C and implements list processing, which manipulates the memory system using pointers to find and sort variables, matrices using common math operations such as the multiply and accumulate instruction, a state machine to evaluate data-dependent branch logic, and a cyclic redundancy check (CRC) to operate XOR gates, shifters, etc.

**Figure 2:**
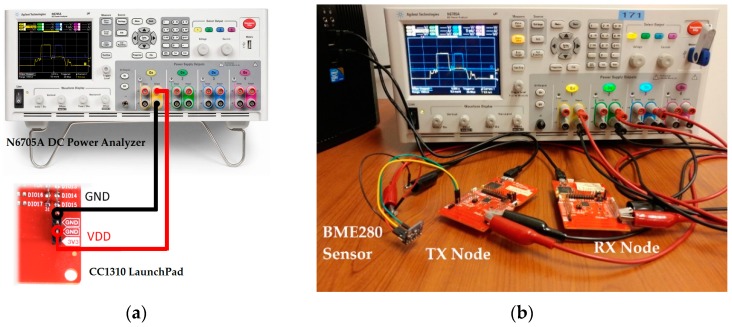
(**a**) Clear cable connections of the (**b**) current consumption measurement setup of the CC1310 wireless MCU while performing a point-to-point communication of an internal temperature sensor data and running the TI 15.4 network stack.

**Figure 3:**
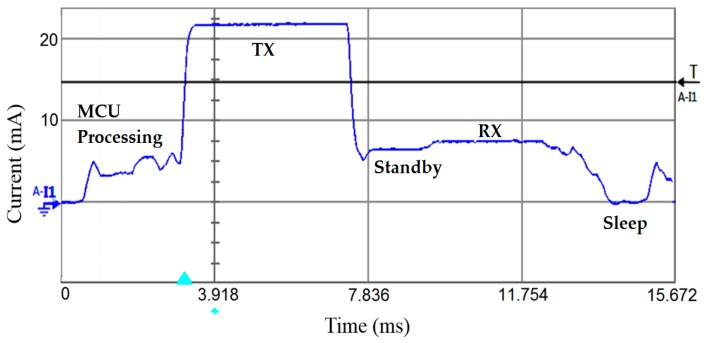
Current consumption profile of the CC1310 wireless MCU on the transmitter’s side.

Table 2 shows a comparison of the average current measurements in [52] to the predictions using CM, showing that the estimation of the average current consumption when using CM results in a 4% error margin, which is acceptable. It should be noted that for the Bluetooth low energy (BLE) current, the contribution of some peripherals has been taken into account and subtracted from the actual measured current in order to determine the 2.825 mA number. This is because CM only evaluates the MCU core. Therefore, it is safe to say that the CM benchmark represents a fairly close workload of a SN and is a reliable indicator of the power consumption of different MCUs. The measurement setup presented in Figure 2 was used to measure the current consumption of the CC1310 wireless MCU that is depicted in Figure 3. The latter shows the processing period, the transmission of the data packet where the highest amount of current is observed, a standby period, and the reception of an acknowledgement (ACK). By using the N6705A DC Power Analyzer from Agilent Technologies, multiple test instruments and external circuitry to analyze the energy requirements of the device under test (DUT) can be omitted. In addition, the “Agilent 14585A Control and Analysis Software” tool is used to control the Agilent N6705A for a better display and control over the equipment.

**Figure 4:**
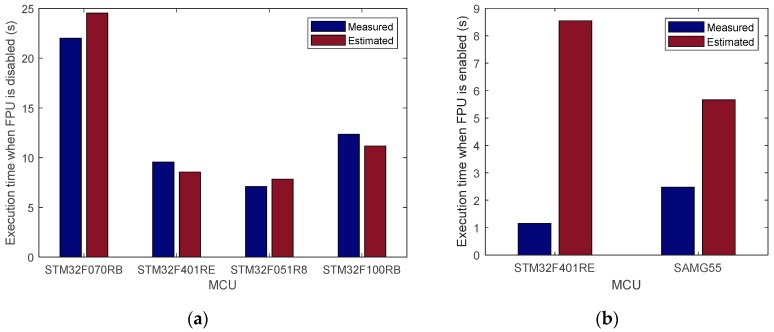
Processing-time estimation using the CM/MHz figure (**a**) when the FPU is disabled and (**b**) when it is enabled.

After estimating the current consumption, an accurate estimation of the processing time is also required in order to evaluate the energy consumption. Metrics such as the million instructions per second (MIPS) are only an approximation as to how a set of processors’ performances would vary, since different amounts of work can be done in one cycle for each processor. Even when using the same intellectual property (IP) core such as the ones provided by ARM, each MCU or system-on-chip (SoC) manufacturer has the freedom to decide whether or not to implement advanced features (e.g., memory accelerators, longer bus fetch widths, floating point unit (FPU)). Figure 4 shows the impact of the FPU on the execution time.

Therefore, rapidly comparing the speed of different MCUs that are becoming more and more complex is not a trivial task. As the CM benchmark becomes an industry standard, the CM/MHz figure is increasingly provided in data sheets. It is judged to be accurate enough to estimate the time it takes different MCUs to process the same workload [53] using

(1) $$t_{PROCESS}=t_{PROC\_REF}\frac{f_{REF}\;S_{REF}}{f_{MCU}\;S_{MCU}},$$

where $t_{proc\_ref}$ and $S_{ref}$ are the reference time and reference CM score, respectively, extracted from the reference MCU. The values $f_{MCU}$ and $S_{MCU}$ are the operating frequency and CM score of the studied candidate MCU and $f_{ref}$ is the reference operating frequency.

It is important to note that the latter can be used when the execution time of the workload on a reference MCU is known. In addition to the aforementioned advantages, CM is judged to be a reliable benchmark because it ensures that compilers would not be able to pre-compute results to completely optimize the work away, unlike the Dhrystone benchmark [53]. Another important characteristic about CM is that results reporting is done following a standard format so they can be eventually certified by EEMBC.

In Figure 4a, the STM32F070RB Cortex-M0 MCU was used as a reference to estimate the processing time of running the room occupancy estimation algorithm [1] on the STM32F401RE Cortex-M4 MCU and vice versa. Additionally, the STM32F051R8 Cortex-M0 MCU was used as a reference to estimate the processing time of the ECDSA cryptography algorithm for IoT applications [6] on the STM32F100RB Cortex-M3 MCU and vice versa. For the four MCUs, the processing time is estimated with an error between 9.4% and 11.5%. Therefore, the estimates are judged to be accurate enough to help make high level decisions. For a better visualization of the data, in Figure 4b, 15,000 iterations of the Temperature-Dependent Kinetic Battery Model (T-KiBaM) algorithm [28], used in battery-powered WSN, are assumed to be running on the SAMG55 32-bit ARM Cortex-M4 MCU. The SAMR21G18A 32-bit ARM Cortex-M0+ MCU studied in [28] was used as a reference. However, when the FPU is enabled, the estimation is no longer acceptable for both MCUs, especially for the STM32F401RE. Figure 4a shows that when the FPU was disabled the time was estimated with a 10.5% error. This is due to the fact that CM primarily focuses on integer operations commonly used in embedded systems and neglects features like the FPU. Moreover, the STM32F401RE is running the room occupancy estimation algorithm [1], which uses a significant number of floating-point operations. In this work, The MCU’s energy consumption is estimated using

(2) $$E_{MCU}=V_{WMCU}\;\left(I_{CoreMark}+I_{PPH}^{MCU}\right)\;t_{process},$$

where $I_{CoreMark}$ is the MCU’s current consumption when running CM, $I_{PPH}^{MCU}$ is the current consumption of other peripherals (e.g., peripheral power domain, RF core, I2C, and timers), and $V_{WMCU}$ is the operating voltage.

#### 2.3.2. Wireless Transceiver and Sensor

**Figure 5:**
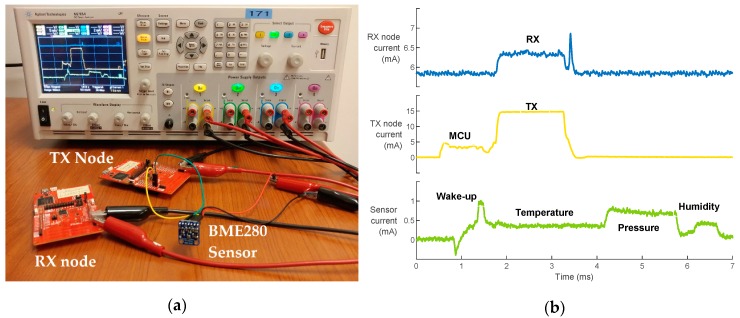
(**a**) Test setup for transceiver and sensor current measurement, and (**b**) current consumption breakdown of the sensor, transmitter, and receiver during one measurement.

To evaluate the energy consumption per measurement of the wireless transceiver, both the transmitter and the receiver are considered. The current measurement setup is shown in Figure 5a.

The studied transceiver uses a sub-1 GHz carrier, which has the capability to respond to the needs and concerns for long-range and low-power wireless connectivity [10,54]. Computing the energy consumption per measurement during the active period is quite straightforward in this model. It can be estimated using

(3) $$E_{TRX}=V_{WMCU}\frac{l}{D_{R}}\left(I_{TX}+I_{RX}\right),$$

where l is the packet length, $D_{R}$ is the data rate, and $I_{TX}$ and $I_{RX}$ are the transmitter’s and the receiver’s currents during active mode, respectively, as shown in Figure 5b.

In this study, the combined digital humidity, pressure, and temperature BME280 sensor from Bosch Sensortec was chosen. It is housed in a compact package, allowing the reduction of the overall sensor node’s form factor. Both the measurement time and the current consumption depend on the oversampling mode of the three physical quantities. This means that, on the sensor’s level, noise can be traded-off against latency and current consumption.

Figure 5b clearly shows the different measurement phases (i.e., temperature, pressure, and humidity, respectively) and current consumption on the BME280 sensor where oversampling factors of 4, 2, and 1 were selected, respectively [50]. It also shows the current consumption profile on the transmitter and receiver. As expected, Figure 3 and Figure 5b show that the transceiver is the most energy-consuming component of a SN. It should be noted that, in this case, the receiver is continuously listening, as depicted in Figure 5b. However, the estimations in this work consider it to be duty-cycled (i.e., only active during the reception period). This is because a simple point-to-point communication is assumed and implemented in this work. Yet, in WSN applications, communication protocols such as time division multiple access (TDMA) are used in order to allow the receiver the go into a power-saving mode when not communicating. Moreover, it can be seen from Figure 5b that the measurement starts right after the MCU’s wake-up and takes a long time to finish. This means that the measurement done in the $i^{th}$ period is actually transmitted in the ${\left(i+1\right)}^{th}$ period. From an energy consumption point of view, the results remain the same. According to the datasheet [50], the sensor’s measurement time can be calculated using

(4) $$t_{SENSOR}=2\;\left(T_{OVS}+P_{OVS}+H_{OVS}+1\right).$$

The average current consumption during measurement can be calculated using

(5) $$I_{SENS}=\frac{I_{DDT}\left(1+2\;T_{OVS}\right)+I_{DDP}\left(2\;P_{OVS}+0.5\right)+I_{DDH}\left(2\;H_{OVS}+0.5\right)}{t_{SENS}}.$$

Therefore, the sensor’s energy consumption is given by

(6) $$E_{SENS}=V_{SENS}\;I_{SENS}\;t_{SENS},$$

where $V_{SENS}$ is the sensor’s supply voltage.

**Figure 6:**
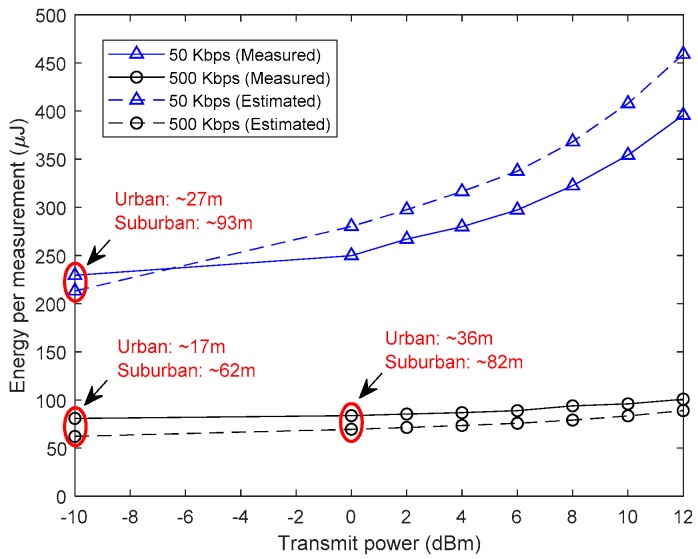
Estimated versus measured SN’s energy per measurement.

In order to see the impact of using different bit rates and output power levels and to evaluate the accuracy of the energy consumption models, measurements were performed at 50 kbps and 500 kbps, with an output power level going from −10 to 12 dBm. The results are depicted in Figure 6. The estimated energy was obtained using Equation (6) for the sensor, Equation (3) for the transceiver, and Equation (2) for the MCU. At this point, it can be assumed from Figure 6 that a better energy efficiency can be achieved with higher data rates, allowing the transceiver to go into a power-saving mode more quickly. However, this remains an assumption as it comes at the cost of a lower communication range. Therefore, the goal is to use the highest achievable data rate that can sustain the desired communication range. The latter is determined for a given bit error rate (BER) or reliability target of the application. To this end, a realistic path loss model is indispensable in order to estimate the received signal power. It is introduced in Section 3.3 of this paper.

## 3. Outdoor Measurements and Wireless Link Characterization

**Figure 7:**
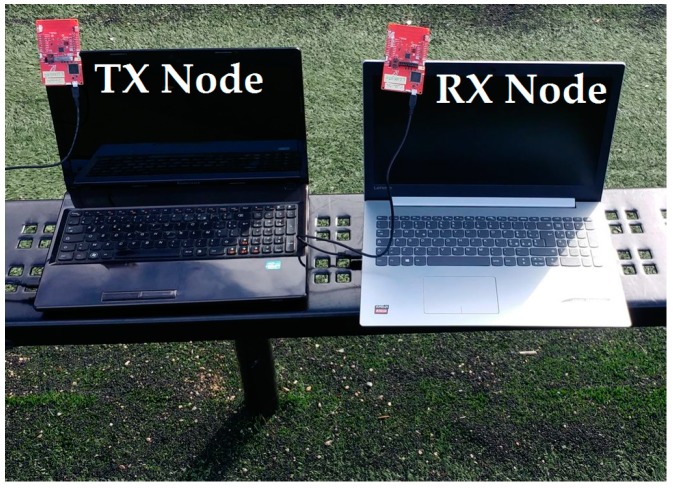
WMCU communication range measurement setup.

Since a SN is intended to operate as part of a big network of other SNs, the energy consumption is considerably affected by the wireless channel condition and the distance between the nodes. Therefore, a realistic model for PL is of interest in order to estimate the received power at the receiver and determine the communication range for a given reliability requirement. To this end, in the following measurements a pair of Sub-1 GHz CC1310 wireless microcontroller unit (WMCU) LaunchPad development kits, operating at 915 MHz, and tow laptops running the SmartRF Studio application [55] are used as depicted in Figure 7. Starting at a 1 m distance, the three following metrics (i.e., PER, BER, and $RSSI_{avg}$) are collected in steps of 5 or 10 meters by keeping the transmitter at the same place and moving the receiver in order to evaluate the radio link quality. The packet error rate (PER) is given by

(7) $$PER=100\;\frac{N_{P}{}_{NOK}+N_{P}{}_{LOST}}{N_{P}},$$

where $N_{P}{}_{NOK}$ represents the number of packets received in error, $N_{P}{}_{LOST}$ is the number of completely lost packets (i.e., the receiver knows that it has to receive a given number of packets), and $N_{P}$ represents the total number of packets, which is in this case 400.

The BER is also taken into account and estimated using

(8) $$BER=100\left(1-{\left(1-\frac{PER}{100}\right)}^{\frac{1}{N}}\right),$$

where N is the number of bits per packet.

Lastly, the RSSI is also considered and calculated using

(9) $$RSSI_{Avg}=\sum_{i=1}^{N_{P}}\frac{RSSI_{i}}{N_{P}},$$

where $RSSI_{i}$ is the received signal strength indicator of the $i^{th}$ packet.

### 3.1. Ambient Noise Density Measurements

**Figure 8:**
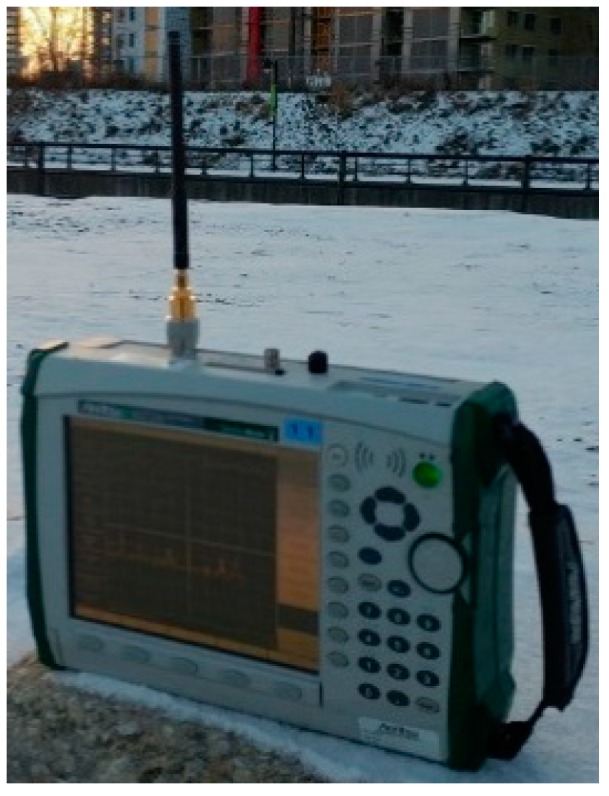
Measurement setup of the ambient noise.

As the sub-1 GHz ISM band is used, interference can considerably affect the wireless link quality and needs to be investigated. Figure 8 shows the measurement setup of the ambient noise density $N_{A}$ by using the MS2721A spectrum analyzer. It is given by

(10) $$N_{A}=k_{b}T+\Delta N,$$

where $k_{b}$ is the Boltzmann constant, T is the ambient temperature, and ΔN is the noise density arising from other interfering emissions in the same frequency band. The spectrum analyzer’s noise marker functionality was used to get a 1 Hz resolution bandwidth measurement. Although the measurement setup is different, the reader can refer to [56] to better understand the measurement methodology. Noise densities of −151.11 dBm/Hz and −154.27 dBm/Hz were measured in the urban and suburban areas respectively. The 3.16 dB difference can be explained by the fact that the urban area is a more industrialized and densely populated area [57].

### 3.2. Communication Range Outdoor Measurements

**Figure 9:**
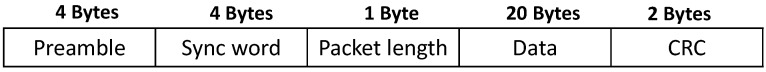
Packet format used in outdoor measurements.

In this section, the link quality characterization of a point-to-point communication link deployed in different outdoor environments (i.e., urban and suburban areas) is presented. Two different output power levels on the transmitter’s side (i.e., −10 dBm and 0 dBm) and two different data rates on the transmitter’s and receiver’s sides (i.e., 50 Kbps and 500 Kbps) are used in order to analyze how link reliability, latency, and energy consumption can be traded-off against each other. The total packet size was set to 31 Bytes, as depicted in Figure 9, and for each measurement 400 packets are sent.

#### 3.2.1. Suburban Area

**Figure 10:**
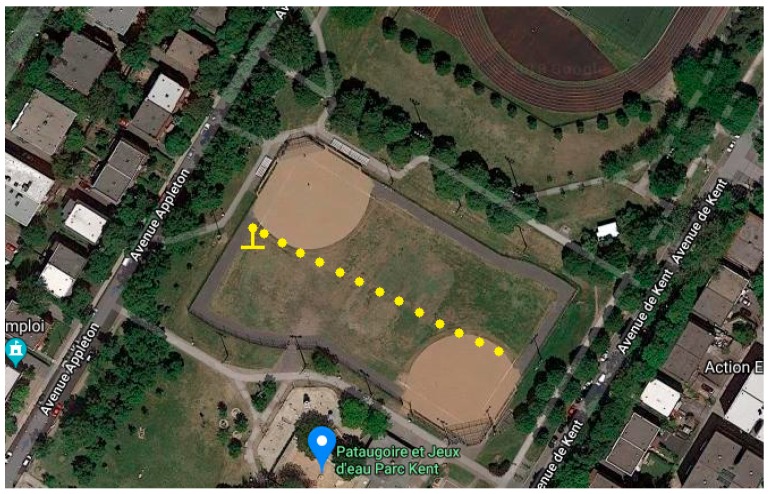
Google satellite image of the field measurement setup in the suburban area.

These measurements were conducted in a residential area in the city of Montreal, in an open baseball field of a public park. The field measurement setup in the suburban area is presented in Figure 10.

#### 3.2.2. Urban Area

**Figure 11:**
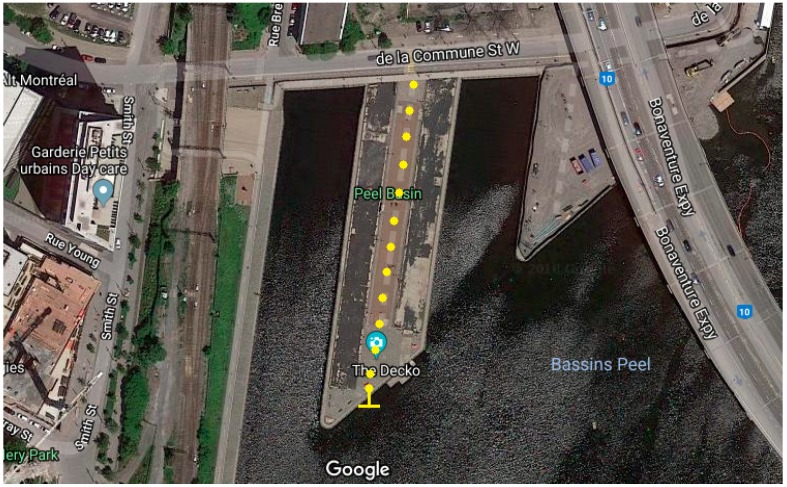
Google satellite image of the field measurement setup in the urban area.

These measurements were conducted in a more dense and industrialized area in the same city, beside a canal not far from downtown. The field measurement setup in the urban area is presented in Figure 11.

#### 3.2.3. Experimental Results

**Figure 12:**
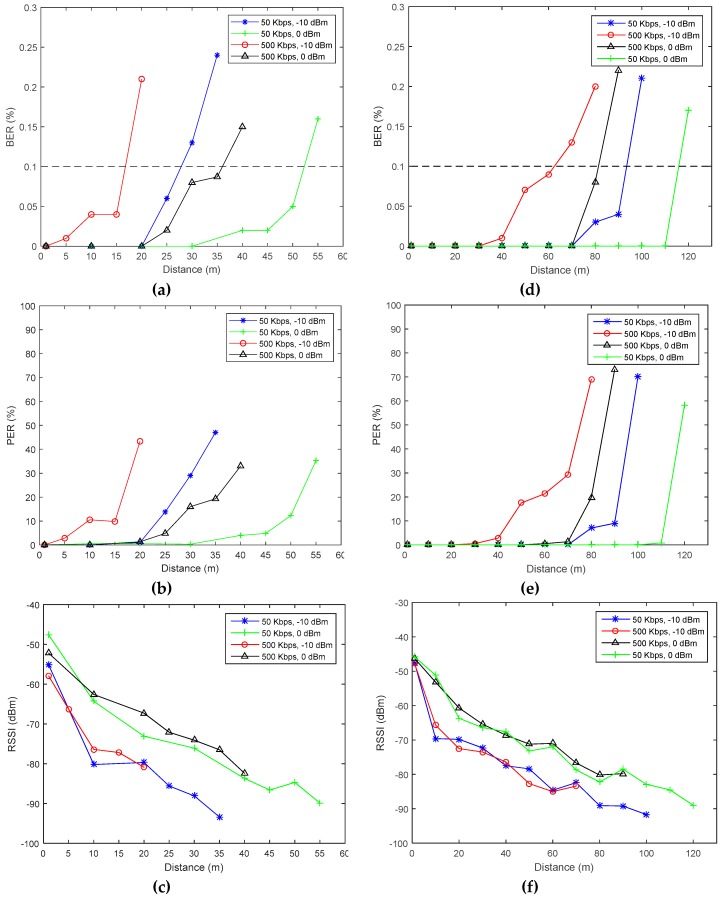
BER, PER, and RSSI field measurements in (**a**–**c**) the urban and (**d**–**f**) the suburban areas.

Figure 12 shows the results of the measurements done in the urban and suburban areas. From these measurement results, it can be seen that increasing the bandwidth, B, in the urban area to achieve a higher data rate has a more pronounced impact on range (i.e., BER≤0.1) when compared with the suburban area, which can be explained by the previously measured higher noise density. The noise power in a given bandwidth is expressed by

(11) $$P_{NOISE}=N_{A}+10\;\log\left(B\right)$$

It was previously mentioned that using high data rates would result in a better energy consumption, as depicted in Figure 6. However, the achievable communication range was yet unknown. Figure 12 shows that, at 0.1% BER as required by Bluetooth applications, similar communication ranges can be achieved by increasing the output power and data rate (i.e., 500 Kbps and 0 dBm) or decreasing them (i.e., 50 kbps and −10 dBm). However, Figure 6 shows that the energy consumption per measurement, when the first configuration is used, is three times less than when using the second. Therefore, the previously made assumption of the need to increase the data rate in order to achieve a better energy efficiency while covering the desired commination range is judged to be logical and valid.

### 3.3. Path-Loss Model

For outdoor applications, the most common multipath signals are caused by ground reflections [11]. The fitting factor proposed in [38] for LOS communications systems is also used with a variable fitting coefficient $l_{f}$. The proposed path-loss model is expressed as follows

(12) $$PL=20\log\left(\frac{4\pi d}{\lambda }\right)-20\log\left[2\sin\left(\frac{2\pi h_{t}h_{r}}{\lambda d}\right)\right]+l_{f}\log\left(50d\right),$$

where d is the distance between the two nodes, λ is the wavelength, $h_{t}$ and $h_{r}$ are the distances that separate the transmitter and the receiver from the ground, respectively, and $l_{f}$ is a fitting coefficient.

**Figure 13:**
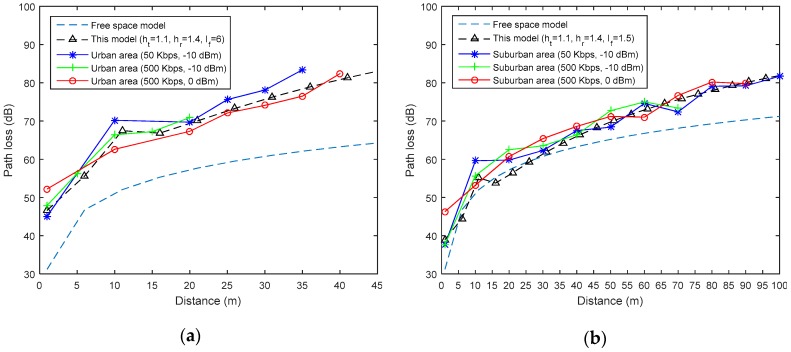
The measured path-loss versus the free-space and estimated ones in the (**a**) urban and (**b**) suburban areas.

Figure 13 shows that the proposed model can accurately estimate the power at the receiver when compared with the free space path loss (FSPL) model. The latter is given by [11],

(13) $$PL=20\log\left(\frac{4\pi d}{\lambda }\right).$$

In the urban area, a fitting coefficient, $l_{f}=6$, was used. However, in the suburban area, a $l_{f}=1.5$ was found to model the path loss more accurately. Moreover, the ground reflection is well-modeled at a distance d≈10 m when the transmitter and the receiver are 1.1 m and 1.4 m away from the ground, respectively. As no antenna gains were considered on the transmitter and receiver sides, the experimental path-loss $PL_{exp}$ was calculated using

(14) $$PL_{exp}=P_{t}-RSSI_{avg},$$

where $P_{t}$ is the output power level. Figure 13 also shows that the FSPL can provide an acceptable estimation of the received power up to 30 m in the suburban area. For longer distances, the FSPL presents an unacceptable error. However, in the urban area, the FSPL model drastically deviates from the experimental results and the proposed model fits.

## 4. Maximizing Data Rate

Now that the received power has been accurately estimated using the proposed path-loss model, the noise detected at the receiver also needs to be accounted for to determine the maximum achievable rate. The signal-to-interference-plus-noise ratio (SINR) needs to be kept sufficiently large to ensure correct demodulation with a given bit error probability threshold required by the application. To this end, the system’s noise power density $N_{sys}$ (i.e., the sum of all unwanted signals that contaminate the signal of interest) in the deployment site needs to be determined. The SINR is given by

(15) $$SINR=\frac{P_{r}}{N_{sys}}=\frac{RSSI}{N_{A}+N_{fg}},$$

where $P_{r}$ and RSS denote the received power and $N_{fg}$ is the noise figure of the receiver. In this case, for the used CC1310 WMCU, $N_{fg}=7\;\mathrm{dB}$ [58].

Using the BER performance curve [59,60] of the used modulation scheme (i.e., Gaussian frequency shift keying (GFSK) in this case), the required energy per bit to noise ratio can be determined to achieve the target BER. Then, using

(16) $${\left[\frac{P_{r}}{N_{sys}}\right]}_{dB}={\left[\frac{E_{b}}{N_{sys}}\right]}_{dB}+{\left[D_{R}\right]}_{dB},$$

the achievable data rate can be determined. An example is included in Section 6.2.

It should be noted that in WSN applications, $D_{R}$. and $P_{t}$. are dynamically changed during operation [10,46]. However, in this work, it is also important to make estimations early in the design process in order to make high-level decisions about the hardware and node configuration to use.

Now that the link parameters are adjusted for a better energy consumption efficiency, communication range and throughput, packet loss needs to be mitigated in order to achieve the required reliability target. Moreover, the latter must be met with latency and energy costs in mind.

## 5. Energy-Latency-Reliability Trade-Off

In WSNs, finding the energy-latency-reliability trade-off is very important, since it captures the interdependence of key parameters from a QoS point of view. The goal of this section is to determine the successful reception probability of a packet when using different data transfer schemes and when at most R transmissions are allowed for each packet. 

### 5.1. Current Consumption Profile of Different Data Transfer Schemes

In this section, the current consumption profile of the transmitter and the receiver are considered when using simple transmissions, convolutional FEC coding, blind retransmissions (BR), and stop-and-wait (SAW) ARQ-based retransmissions. 

**Figure 14:**
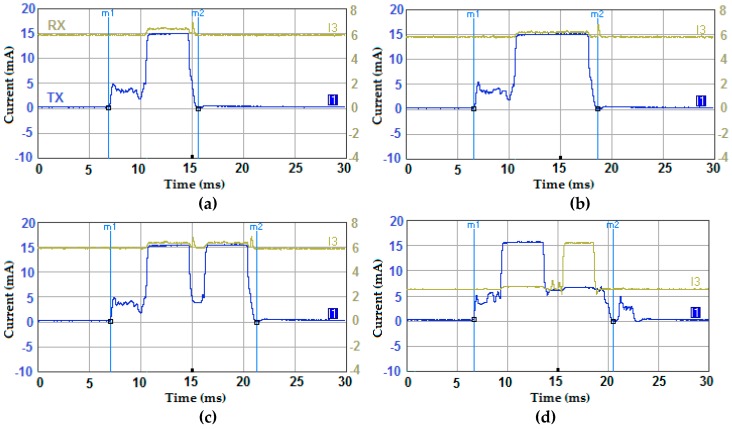
Current consumption profile of the transmitter and the receiver when using different data transfer schemes; (**a**) simple transmissions, (**b**) FEC, (**c**) two BR, and (**d**) a SAW-ARQ protocol (also referred to as 1-Rep-ACK in this work).

The same measurement setup in Figure 2 was used and the results are depicted in Figure 14. Figure 14b shows a considerable transmission time and energy overheads when using FEC as the payload is doubled due to coding when compared with Figure 14a. Similarly, transmitting the packet twice as shown if Figure 14c or waiting for an ACK as depicted in Figure 14d has more pronounced energy and time overheads.

**Figure 15:**
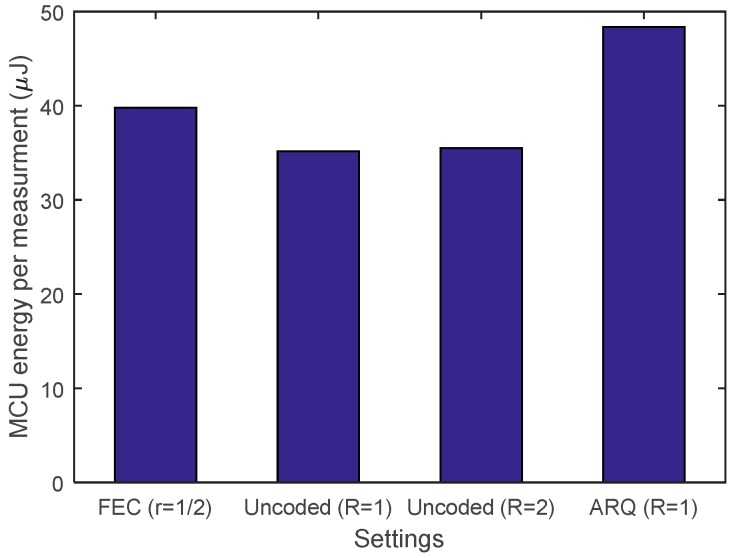
MCU energy consumption per measurement for different data transfer schemes.

It should be noted that, apart from the transceiver overhead, Figure 15 shows that the MCU energy consumption per measurement does vary when using different data transfer schemes. An energy overhead can be observed when using FEC and the SAW-ARQ protocol due to additional data encoding and packet processing, respectively. However, when compared to the transceiver’s contribution which is investigated in two case studies presented in Section 6.2, it is safe to say that the MCU’s energy consumption does not make a significant difference.

All of the previously mentioned techniques (i.e., FEC, BR, and SAW-ARQ) can manage bit errors. Yet, the packet success probability needs to be determined for each technique in order to objectively investigate their usefulness.

### 5.2. Packet Success Probability

In order to fairly compare the three different data transfer schemes from an energy, latency, and reliability point of view simultaneously, as will be discussed in Section 6.2, the packet success probability must be estimated first. In the probabilistic binary symmetric channel (BSC) model, a bit is independently flipped with a bit error probability $P_{e}$ during transmission. If node A is sending packets to node B through a BSC, where all packets have the same size of N bits, the probability that a packet from A arrives error-free at B is given by

(17) $$P={\left(1-P_{e}\right)}^{N}.$$

This probability can be enhanced by using error correction techniques. Namely, cases of FEC, BR, and SAW-ARQ retransmissions are investigated in this work.

#### 5.2.1. Forward Error Correction (FEC)

FEC is being widely used to cope with the random erroneous bits in a data packet. As an example, a convolutional encoder (n, k, K) is implemented by adding n−k redundant bits to the actual k bits of data. The number of bits upon which the encoder’s output depend, K, is called the constraint length or depth of the code. The ratio $r=\frac{k}{n}$ is called the code rate. The performance of a convolutional code is also characterised by its free distance, $d_{m}$, which is the minimal hamming distance [61] between different encoded sequences. This means that changing one bit in the message sequence will change at least $d_{m}$ bits in the coded output sequence [62].

**Figure 16:**
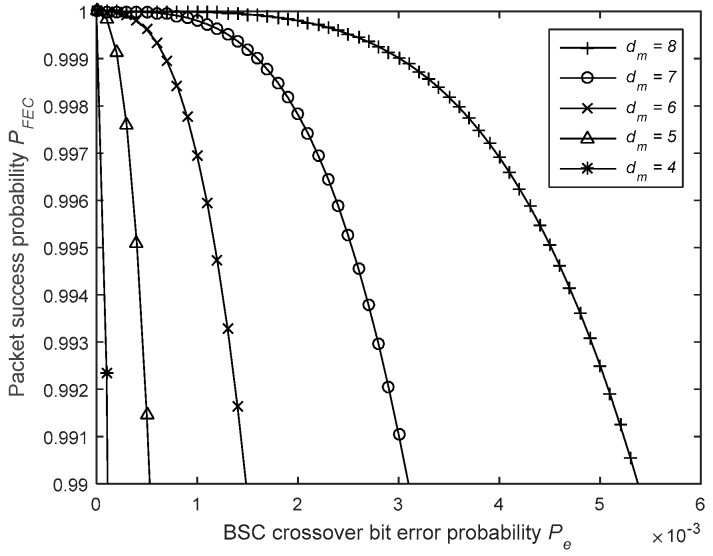
Impact of the free distance $d_{m}$ on the packet success probability.

Figure 16 shows the impact of the free distance on the performance of the code. The asymptotic coding gain that can be achieved can be expressed as follows [62]:

(18) $$G_{FEC}=10\;\log\left(d_{m}\;r\right).$$

**Figure 17:**
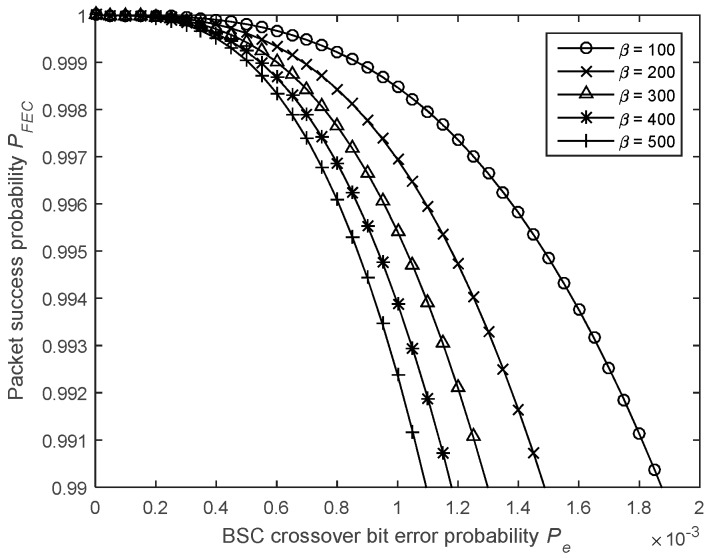
Impact of the $\beta_{free}$ parameter on the packet success probability.

Therefore, the used code ($d_{m}=6$, $r=\frac{1}{2}$) has a theoretic asymptotic coding gain of 4.77 dB. However, the real achievable gain is considerably less and it is generally between 2 and 3 dB [63]. In this case study, a convolutional code with a rate of $r=\frac{1}{2}$, a constraint length of K=4, and free distance at $d_{m}=6$ is considered [62]. Moreover, the error correcting ability of a code depends on the decoding method. The probability that a bit will be received in error when using convolutional FEC and Viterbi decoding, without considering the influence of an interleaver to cope with burst errors, can be approximated using [41]

(19) $$P_{e}^{FEC}\approx \frac{\beta_{free}}{k}{\left(2\sqrt{P_{e}\left(1-P_{e}\right)}\right)}^{d_{m}}\approx \frac{\beta_{free}}{k}2^{d_{m}}P_{e}^{\frac{d_{m}}{2}},$$

where $\beta_{free}$ is the total number of non-zero information bits of all paths with a weight of $d_{m}$. It should be noted that $\beta_{free}$ depends on the data pattern and is totally random. Figure 17 shows the impact of $\beta_{free}$ on the performance of the error correcting code. For the sake of simplicity, in this study, $\beta_{free}$ was fixed at 200, which is logical when using payloads of 160 bits. In this case, the probability that a packet from A arrives error-free at B is given by

(20) $$P_{FEC}={\left(1-P_{e}^{FEC}\right)}^{N}.$$

When FEC is used, the required time to send a packet is given by

(21) $$t_{TRX}^{FEC}=\frac{l^{FEC}}{D_{R}},$$

where $l^{FEC}$ is the packet length when using FEC. Therefore, the energy consumption of the transceiver can be expressed as follows:

(22) $$E_{TRX}^{FEC}=V\;t_{TRX}^{FEC}\;\left(I_{TX}+I_{RX}\right).$$

#### 5.2.2. Blind Retransmissions (BR)

In this case, node A blindly transmits R times the packet with a packet successful reception probability P over each transmission. The number of successful packet transmissions h after R independent transmission trials is a random variable S that follows the binomial distribution with parameters R ∈ ℕ and P∈[0,1] and is given by the probability mass function

(23) $$f\left(h,R,P\right)=pr\left(S=h\right)=\left(\begin{array}{c} R\\ h \end{array}\right)P^{h}{\left(1-P\right)}^{R-h}.$$

In accordance with [64,65], the probability that at least one packet is received successfully within R independent attempts can be calculated using

(24) $$P_{R}=1-pr\left(S=0\right)=1-{\left(1-P\right)}^{R}=1-{\left(1-{\left(1-P_{e}\right)}^{N}\right)}^{R}.$$

**Figure 18:**
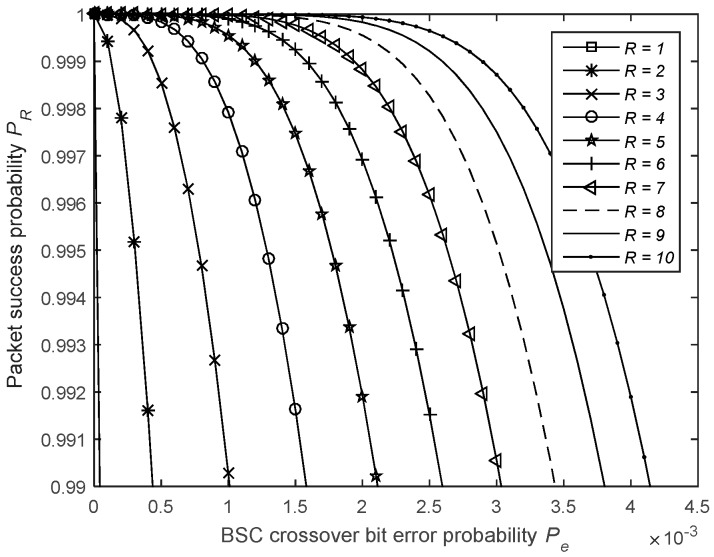
Impact of the number of blind transmission attempts on the packet success probability.

Figure 18 shows the impact of R on the packet success probability. When using BR, the required time to send a packet is given by

(25) $$t_{TRX}^{R}=R\frac{l}{D_{R}}+t_{id}\left(R-1\right),$$

where $t_{id}$ is the time spent in idle mode between two packet transmissions. Therefore, the energy consumption of the transceiver can be expressed as follows

(26) $$E_{TRX}^{R}=V\left[R\frac{l}{D_{R}}\left(I_{TX}+I_{RX}\right)+2I_{id}t_{id}\left(R-1\right)\right].$$

#### 5.2.3. ARQ-Based Retransmissions

**Figure 19:**
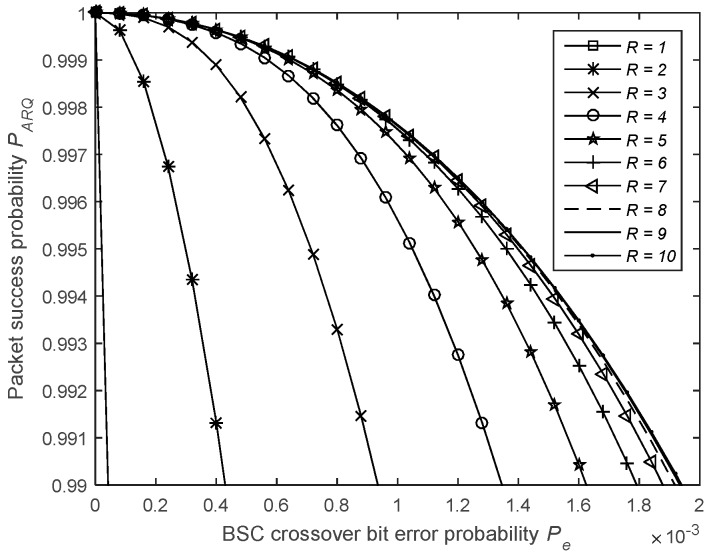
Impact of the number of 1-Rep-ACK transmission attempts on the packet success probability.

In this section, the notation $\bar{z}$ is used to denote $\bar{z}=1-z$, where the real valued variable z∈[0,1]. In this analysis, the *L*-Rep-ACK [39] protocol, which achieves a better feedback channel reliability by retransmitting ACK/NACK packets, is considered. Case studies presented in Section 6 include examples and discussions about the matter. The transmission is considered successful only if the transmitter receives L ACK packets for one data packet where L>1. It should be noted that the particular case where L=1 is the regular SAW-ARQ protocol. The same bit error probability, $P_{e}$, during transmission of ACK and non-acknowledgement (NACK) packets of $N_{f}=40$ bits through the feedback channel is considered. The probability of successfully receiving a data packet at B after a maximum of R *L*-Rep-ACK transmissions is given by

(27) $$P_{R}^{L-Rep-ACK}=1-P_{out}^{L-Rep-ACK},$$

where L is the time diversity order (i.e., number of ACK/NACK transmissions) and $P_{out}^{ARQ}$ is the outage probability [39] of the *L*-Rep-ACK protocol and given by

(28) $$P_{out}^{L-Rep-ACK}=\sum_{j=1}^{R-1}{\bar{P}}^{j}P_{pe}^{L}{\left(1-P_{pe}^{L}\right)}^{j-1}+{\bar{P}}^{R}{\left(1-P_{pe}^{L}\right)}^{R-1},$$

where j is the index of the transmission attempt and $P_{pe}={\bar{P}}_{f}=1-{\left(1-P_{e}\right)}^{N_{f}}$ is the failure probability of an ACK packet. Figure 19 shows the impact of the maximum allowed number of 1-Rep-ACK transmission attempts, R, on the packet success probability.

**Figure 20:**
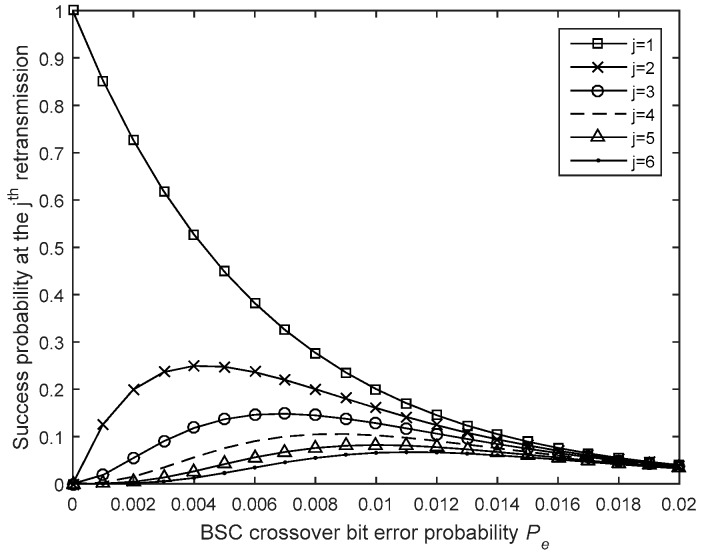
Packet success probability at the $j^{th}$ packet.

Moreover, as illustrated in Figure 20, using a maximum of R ARQ transmissions does not mean that R is always reached and that is the reason why it is possible to achieve better energy and latency efficiencies when using acknowledgements. The probability that a packet will be received successfully at the $j^{th}$ packet transmission attempt is given by

(29) $$P_{j}^{L-Rep-ACK}=P_{R}^{L-Rep-ACK}\times {\left(1-P_{R}^{L-Rep-ACK}\right)}^{j-1}.$$

When using *L*-Rep-ACK retransmissions, the required time to send a packet is given by

(30) $$t_{TRX}^{ARQ}=\overset{R}{\underset{j=1}{\sum }}P_{j}^{ARQ}\left[j\left[\left(\frac{l_{data}}{D_{R}}+\frac{L\;l_{ack}}{D_{R}}\right)+L\;t_{sb}\right]+t_{id}\left(j-1\right)\right],$$

where $t_{sb}$ is the time spent in standby mode after sending the packet and before receiving the acknowledgement and $l_{ack}$ is the length of the acknowledgement packet. Therefore, the energy consumption of the transceiver can be expressed as follows

(31) $$E_{TRX}^{ARQ}=V\sum_{j=1}^{R}P_{j}^{ARQ}\left[j\left[\left(\frac{l_{data}}{D_{R}}+\frac{L\;l_{ack}}{D_{R}}\right)\left(I_{TX}+I_{RX}\right)+2\;L\;I_{sb}t_{sb}\right]+2I_{id}t_{id}\left(j-1\right)\right].$$

Using the previous analytical results, the three reliable data transfer schemes can be objectively compared while taking into account the achievable reliability, the energy, and latency costs.

## 6. Resulting Design Methodology and Case Studies

This section explains how all of the previous steps are logically linked together to form the system-level design methodology. Moreover, case studies and examples further demonstrate how the methodology can be applied.

### 6.1. Design Flow and Methodology

**Figure 21:**
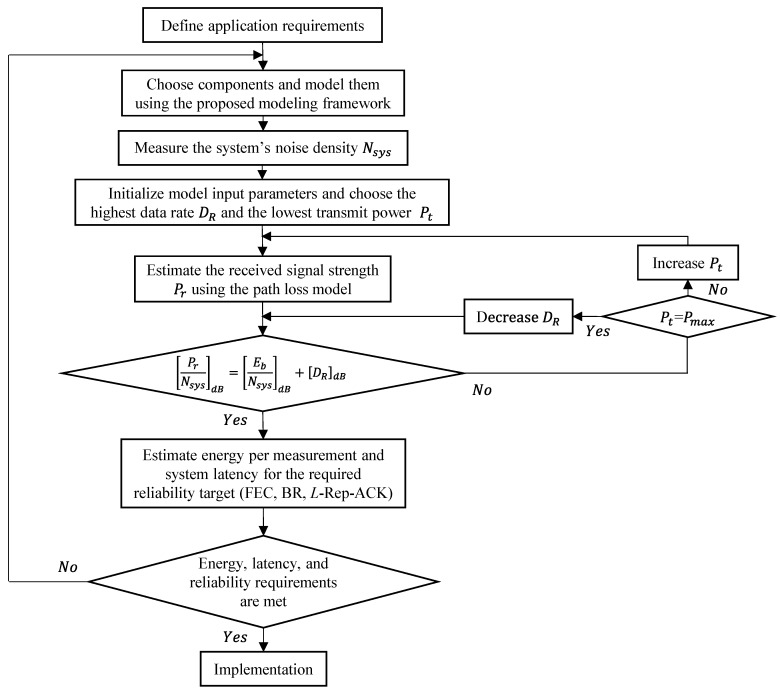
SN design flow graph.

Early in the design process, a SN designer would follow the steps shown in the design flow graph presented in Figure 21 in order to make high-level decisions about the right COTS components to use and the possibility to meet the reliability, energy, and latency requirements. At this point, all of the model inputs, which are outlined in Table 1, should be available. Then, the designer needs to determine the system’s noise density by measuring the background noise in the deployment site and taking into account the receiver’s noise figure. Before estimating the energy consumption per measurement, it should be made sure that an acceptable communication range can be achieved. Therefore, starting by using the highest data rate and the lowest output power level on the transceiver, the designer estimates the range and iterates between the two parameters while always maximizing the data rate. Once a good communication range is achieved for a given BER target value, the energy consumption per measurement while using different reliable data transfer schemes is estimated. Finally, given the application requirements, if a good trade-off between energy, latency, and reliability is found, the designer would proceed with the implementation phase. Otherwise, other COTS components need to be selected.

### 6.2. Case Studies

Considering the measurements reported in Section 3, the example illustrated in Table 3 shows how to estimate the maximum achievable data rate when the input parameters are known. 

First, the received power, $P_{r}$, is estimated using the PL model considered at the targeted range. Second, the SINR is determined. Finally, the achievable data rate is calculated. In this case, the latter is estimated to be around 44.668 kbps. However, when compared to the measurements reported in Figure 12b, an error of 10.66% is observed as the actual data rate is 50 kbps. This is acceptable as noise and signal attenuation do vary over time. Therefore, in WSN applications, $D_{R}$ and $P_{t}$ are dynamically changed during operation [10,46]. Theoretically, increasing $P_{t}$ by 10 dBm (i.e., $P_{t}=0\;\mathrm{dBm}$) would result in achieving the same range at 500 kbps and, therefore, lowering the energy per measurement from 213 μJ to 62 μJ, as shown in Figure 6. However, practically, the measurements in Figure 12b show that the range becomes around 80 m which is 15 m shorter (i.e., a 15.8% range reduction) than the theoretically estimated range of 95 m. This can be tackled by further increasing $P_{t}$, which will always be a better compromise for the CC1310 radio chip in terms of latency and energy consumption as shown in Figure 6. Moreover, it is safe to say that this is also true for a wide range of current generation and similar wireless transceivers in the market.

#### 6.2.1. 99% Reliability Target

Like in [7], the reliability in this work is directly linked to the packet success probability, P. Therefore, in order to evaluate the data transfer reliability of a point-to-point link, an application’s requirement of 0.99 in terms of packet success probability is assumed, which corresponds to a 99% reliability figure of merit. This level of reliability is required by applications, such as utility-to-consumer real time pricing, outage management, and automated feeder switching in a smart grid [5,66]. Also, a forward and feedback channel bit error probability, $P_{e}=10^{-3}$, is assumed.

**Figure 22:**
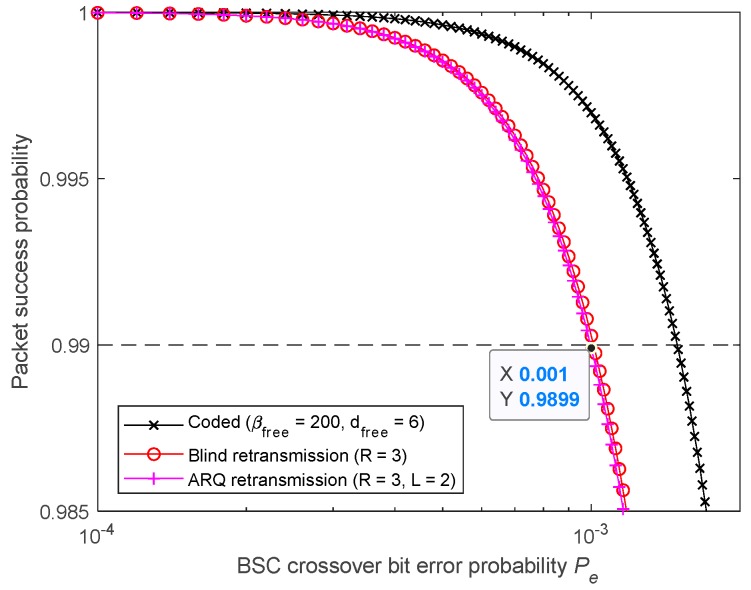
Meeting 99% reliability target by using FEC, BR, and 2-Rep-ACK retransmissions (*L* = 2).

**Figure 23:**
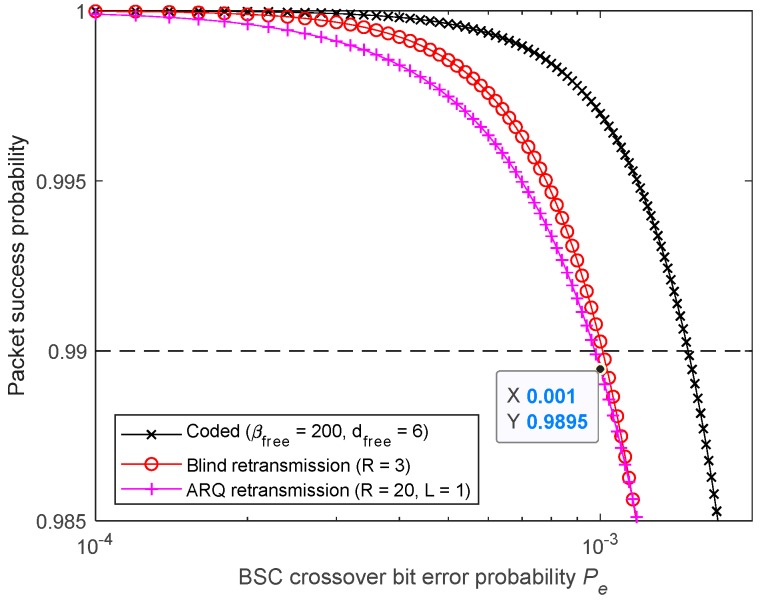
Meeting 99% reliability target by using FEC, BR, and 1-Rep-ACK retransmissions (*L* = 1).

Figure 22 Shows that the requirement is met by the studied FEC code. When using BR, only two retransmissions are needed (R=3). However, the 2-Rep-ACK approach, which is also affected by the imperfect feedback channel, requires three retransmissions (R=4), as two retransmissions (R=3) are not enough. In fact, for a feedback channel having the same packet error probability as the forward’s (L=1), a large number of 1-Rep-ACK transmissions (i.e., R>20) is required, as shown in Figure 23. As such, for identical forward and feedback error probabilities, it is appropriate to consider *L* > 1 for the ACK transmissions.

The overall energy consumption per measurement $E_{sys}$ is given by

(32) $$E_{sys}=E_{TRX}+E_{MCU}+E_{SENSOR}.$$

Moreover, the latency of the wireless link $t_{sys}$ can be calculated using

(33) $$t_{sys}=t_{TRX}+t_{PROCESS}+t_{SENSOR}.$$

**Figure 24:**
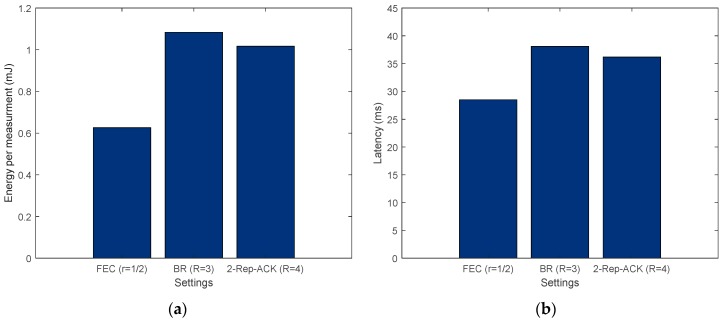
(**a**) Energy consumption per measurement and (**b**) system latency for 99% reliability target.

The energy per measurement results presented in Figure 24a were obtained using Equation (32). It is assumed that the sensor’s and MCU’s energies are not changed from one data transfer scheme to another. Moreover, the results in Figure 24b were obtained using Equations (33). Both figures show that when the required reliability is around 99%, using FEC is the least expensive choice, in terms of energy consumption and latency.

Therefore, by way of example, without applying this methodology, a designer could have used the regular SAW-ARQ and not met the required reliability. Moreover, if BR were arbitrarily used (R = 3) to ensure the 99% reliability requirement, the energy consumption and latency would increase by around 75% and 32%, respectively, when compared with opting for FEC.

#### 6.2.2. 99.999% Reliability Target

A requirement of 0.99999 in terms of packet success probability is now assumed, which corresponds to a 99.999% reliability figure of merit. This level of reliability is required by applications such as industrial IoT [67,68,69] and wide area situation awareness [7]. Additionally, a forward and feedback channel bit error probability $P_{e}=10^{-3}$ is assumed.

**Figure 25:**
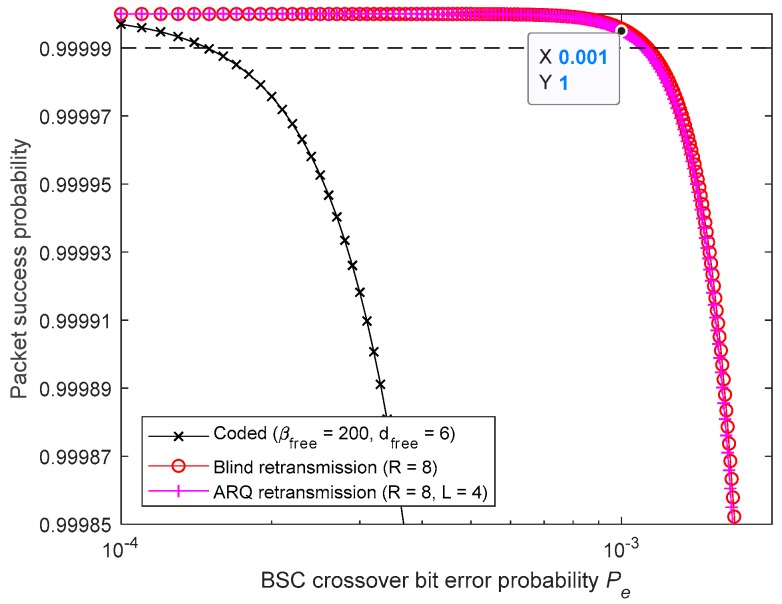
Meeting 99.999% reliability target by using BR and 4-Rep-ACK retransmissions.

**Figure 26:**
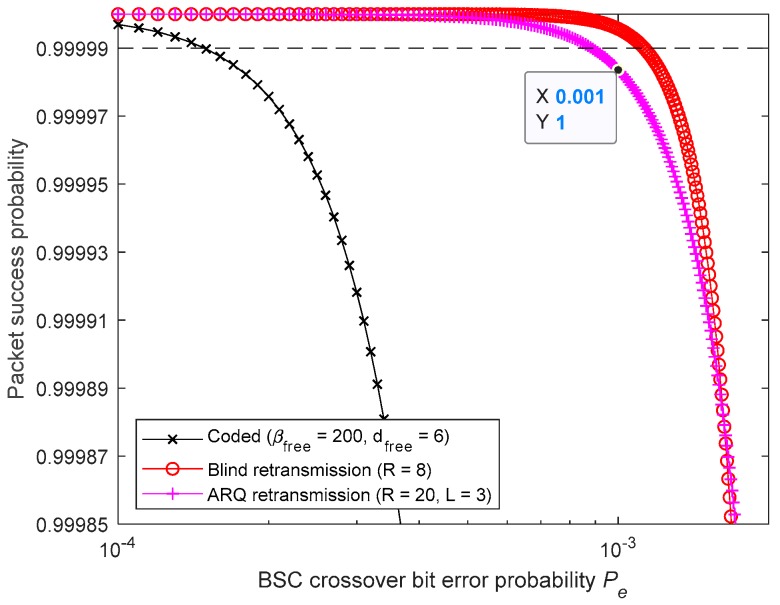
Meeting 99.999% reliability target by using BR and 3-Rep-ACK retransmissions.

Figure 25 and Figure 26 show that the requirement is no longer met by the studied convolutional FEC code. Therefore, a code presenting a longer free distance, $d_{m}$, is required. Furthermore, it can be concluded from Figure 25 that, when using BR, seven retransmissions are needed (R=8) and, when using 4-Rep-ACK, seven retransmissions are required (R=8). However, for the latter, that can be achieved only when L≥4. Figure 26 shows that with L=3, a large number of 3-Rep-ACK transmissions (i.e., R>20) are required, making this L value unviable.

**Figure 27:**
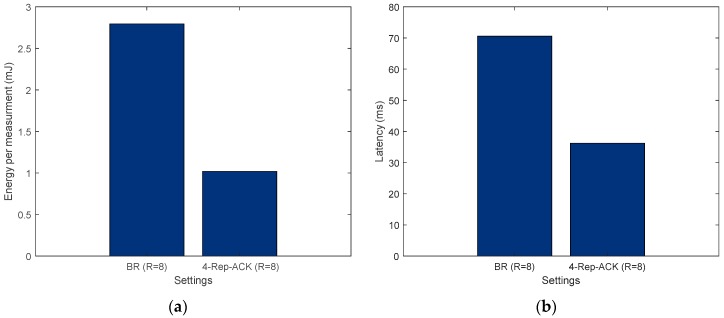
(**a**) Energy consumption per measurement and (**b**) system latency for a 99.999% reliability.

Figure 27 shows that, in this case, using 4-Rep-ACK retransmissions is the least expensive choice in terms of energy consumption and latency, provided that the feedback channel is significantly more reliable (i.e., L=4).

It can be concluded from these case studies that, for SAW-ARQ, a noisy feedback channel can severely degrade system performance and make it worse than an open-loop system (i.e., BR approach) [70]. However, when the feedback channel is much less error-prone, the SAW-ARQ protocol can be as reliable as when using the time and energy inefficient BR. However, in WSN applications, this is not the case and the feedback channel is also error-prone with similar probabilities [70,71]. For this reason, ACK-NACK responses have to be sent with a stricter reliability requirement in mind, leading to a larger value of the L requirement for the ACK approach [72,73].

## 7. Conclusions

In this paper, a methodology to design, configure, and deploy a reliable ultra-low power WSN was proposed. It can yield a better energy efficiency, latency, and reliability. Therefore, a comprehensive energy model of the sensor node, along with a modeling framework, were presented and validated through measurements. In addition, a realistic PL model was presented for both urban and suburban areas and based on field measurements. Then, after measuring the noise, the achievable data rate was determined in order to ensure energy efficiency and short latency. Furthermore, in order to mitigate wireless transmission errors, three error correcting techniques were studied and compared in terms of energy consumption, latency, and reliability. 

Based on the model and analysis presented, a methodology which logically links the comprehensive design and deployment steps of an ultra-low power and reliable WSN was also presented and detailed.

By using estimations and measurements, it was shown that, following the proposed methodology, the designer can thoroughly explore the design space, make the most favorable decisions when selecting SN components, and efficiently configure and deploy a WSN while taking into account the energy-reliability-latency trade-off of different error correction techniques. Through case studies, it was demonstrated how energy, latency, and reliability are interrelated and traded-off against each other, notably with respect to successful packet transmission probability metrics. 

Therefore, the outcomes of this paper can have a significant impact on the design of WSN in a wide range of energy and latency conscious applications. Whether in smart city, precision agriculture, or other monitoring and control applications, the proposed models and methodology can lead to a substantial improvement of a network’s lifetime while recognizing and meeting QoS requirements.

## Figures and Tables

**Table 1:** Energy model parameters.

Parameter	Description	Purpose
$V_{WMCU}$	Operating voltage of the SoC	Power/Energy consumption
$V_{SENS}$	Operating voltage of the sensor	Power/Energy consumption
$D_{R}$	Data rate	TX and RX active times
$f_{MCU}$	MCU operating frequency	Active current/Processing time
$I_{STATE}^{TRX}$	Current consumption in each state	Power/Energy consumption
$I_{STATE}^{MCU}$	Current consumption in each state	Power/Energy consumption
$I_{PPH}^{MCU}$	Peripheral current consumption	Power/Energy consumption
l	Packet length	Transceiver active time
$S_{REF}$	Reference CoreMark score	Processing time/System latency
$f_{REF}$	Reference operating frequency	Processing time/System latency
$t_{PROC\_REF}$	Reference processing time	Processing time/System latency
$S_{MCU}$	Selected MCU’s CoreMark score	Processing time/System latency
$T_{ovs}^{*}$	Temperature oversampling factor	Current consumption/System latency
$H_{ovs}^{*}$	Humidity oversampling factor	Current consumption/System latency
$P_{ovs}^{*}$	Pressure oversampling factor	Current consumption/System latency
$I_{STATE}^{SENSOR}$	Current consumption in each state	Power/Energy consumption

* These parameters are specific to the BME280 sensor used in this work.

**Table 2:** Accurate current consumption estimation using CM.

MCU	Software	Measured Current (mA)	CM Current (mA)	Error (%)
CC1310	TI 15.4 stack	3	2.88	−4
CC2650/CC2640R2	BLE stack	2.825	2.938	4

**Table 3:** Example explaining how to estimate the achievable data rate.

	Parameter	Value
**Inputs (based on data sheet information, measurements, and application requirements)**	$N_{A}$	−154.27 dBm/Hz
	$N_{fg}$(CC1310) [58]	7 dB
	*BER* (requirement)	0.1%
	$P_{t}$	−10 dBm
	*d* (requirement)	95 m
	$\frac{E_{b}}{N_{sys}}$ (at 0.1% *BER*) [59,60]	10.3 dB
	*PL* (This model at 95 m)	80.5 dB
**Results**	$P_{r}$ (Equation (14))	−90.5 dBm
	*SINR* (Equation (15))	56.77 dBm
	$D_{R}$ (Equation (16))	44.668 kbps
